# Assessment of Factors Contributing to Multidrug Resistance in Urinary Tract Infections: Focus on Carbapenem Resistance

**DOI:** 10.3390/antibiotics14090918

**Published:** 2025-09-11

**Authors:** Carina Alexandra Bandac, Constantin Ristescu, Pavel Onofrei, Ionela-Larisa Miftode, Rodica Radu, Vasile Lucian Boiculese, Ana-Maria Raluca Pauna, Theodor Florin Pantilimonescu, Andreea Luduşanu, Viorel Dragoș Radu

**Affiliations:** 1 Department of Urology and Renal Transplantation, "C.I. Parhon" University Hospital, 700115 Iasi, Romania; carina_bandac@email.umfiasi.ro (C.A.B.); ristescu.constantin@umfiasi.ro (C.R.); pantilimonescu.theodor-florin@d.umfiasi.ro (T.F.P.); viorel.radu@umfiasi.ro (V.D.R.); 2 Department of Urology, Elytis Hospital, 700010 Iasi, Romania; onofrei.pavel@umfiasi.ro; 3 Department of Morpho-Functional Sciences II, Faculty of Medicine, University of Medicine and Pharmacy “Gr. T. Popa”, 700115 Iasi, Romania; 4 Department of Infectious Diseases, Faculty of Medicine, University of Medicine and Pharmacy “Grigore T. Popa”, 700115 Iasi, Romania; 5 “St Parascheva” Clinical Hospital of Infectious Diseases, 700116 Iași, Romania; 6 Department of Internal Medicine, Faculty of Medicine, University of Medicine and Pharmacy “Grigore T. Popa”, 700115 Iasi, Romania; rodica.radu@umfiasi.ro; 7 Department of Preventive and Interdisciplinarity, Medical Informatics and Biostatistics, Faculty of Medicine, University of Medicine and Pharmacy “Grigore T. Popa”, 700115 Iasi, Romania; vasile.boiculese@umfiasi.ro; 8 Department of Anatomy, Faculty of Medicine, University of Medicine and Pharmacy “Grigore T.Popa”, 700115 Iasi, Romania; ana-maria.pauna@umfiasi.ro; 9 Department of Morphofunctional Sciences I—Anatomy, University of Medicine and Pharmacy “Gr. T. Popa”, 700115 Iasi, Romania; andreea.ludusanu@umfiasi.ro; 10 Department of Urology, Faculty of Medicine, University of Medicine and Pharmacy “Grigore T. Popa”, 700115 Iasi, Romania

**Keywords:** carbapenem-resistant Enterobacterales, urinary tract infections, multidrug-resistant organisms, antibiotic resistance, carbapenemase-producing bacteria, healthcare-associated infections, antimicrobial stewardship

## Abstract

Introduction: Urinary tract infections (UTIs) caused by carbapenem-resistant pathogens are increasingly common and pose serious treatment challenges due to limited antibiotic options and high complication rates. Identifying patients at risk is essential for guiding empirical therapy and improving outcomes. The primary objective of this study was to identify risk factors associated with carbapenem-resistant (CR) UTIs by comparing them with carbapenem-susceptible (CS) UTIs. Secondary objectives included analyzing the types of microorganisms involved in both groups, their antibiotic susceptibility profiles, and the presence of carbapenemase enzymes among CR UTI cases. Method: We conducted a retrospective case-control study involving 127 hospitalized patients with UTIs caused by CR microorganisms and 91 patients with UTIs caused by multidrug-resistant (MDR) strains that retain susceptibility to carbapenems, admitted between 1 October 2023, and 31 March 2025. Results: In univariate analysis, CR UTI patients had significantly higher rates of neoplasia, neurological disorders, urosepsis at admission, septic shock, the presence of urinary catheters at diagnosis, permanent nephrostomy catheters, hospitalizations within the past 180 days, previous antibiotic exposure including carbapenems, and recent urological procedures. Multivariate analysis revealed four independent risk factors for CR UTIs: neoplasia (OR = 2.152; 95% CI: 1.044–4.436; *p* = 0.038), neurological disorders (OR = 7.427; 95% CI: 2.804–19.674; *p* < 0.0001), antibiotic use in the previous 180 days (OR = 2.792; 95% CI: 1.487–5.396; *p* = 0.001), and prior carbapenem treatment OR = 10.313; 95% CI: 1.277–83.248; *p* = 0.029). Most of the isolated organisms belonged to the Enterobacterales genus, with *Klebsiella* spp. and *Pseudomonas aeruginosa* being the most common pathogens in CR UTIs, accounting for over 90% of cases. Among patients tested for carbapenemase production, all but one tested positive for at least one carbapenemase. Conclusions: Neoplasia, neurological disorders, recent antibiotic therapy, and prior carbapenem use were significantly associated with increased risk of developing CR UTIs. *Klebsiella* spp. and *Pseudomonas aeruginosa* were the predominant causative organisms, with New Delhi metallo-β-lactamase (NDM) and *Klebsiella pneumoniae* carbapenemase (KPC) being the most frequently identified resistance mechanisms.

## 1. Introduction

Urinary tract infections (UTIs) rank among the most prevalent infectious diseases globally, exerting a significant burden on patient health and healthcare systems due to treatment costs and the management of associated complications [1]. Over the past few decades, the growing threat of antibiotic resistance has turned previously uncomplicated UTIs into complex clinical challenges, with serious implications for medical practice and public health [2,3]. Of particular concern are infections caused by multidrug-resistant (MDR) bacteria—defined as strains exhibiting resistance to at least one agent in three or more antimicrobial categories [4].

A recent study estimated that UTIs were directly responsible for approximately 22,000 deaths in the United States in 2019 [1], and this number is expected to rise as urinary pathogens continue to develop resistance to commonly used antibiotics. The diminishing effectiveness of available therapies, combined with the adverse effects of antibiotics on the host microbiome, underscores the urgent need for alternative treatment strategies [5]. Carbapenems, often regarded as ’last-resort’ antibiotics, are valued for their broad-spectrum activity—particularly against extended-spectrum β-lactamase (ESBL)-producing Gram-negative bacilli and some Gram-positive cocci. However, they have limited efficacy against certain multidrug-resistant organisms, including carbapenemase-producing strains, and are generally ineffective against most methicillin-resistant *Staphylococcus aureus* and vancomycin-resistant *Enterococcus faecalis*.

However, resistance to carbapenems is driven by a multifaceted array of mechanisms. The most prominent among these is the production of carbapenemase enzymes—such as KPC, NDM, OXA, and VIM—which hydrolyze carbapenems and render them ineffective. In *Klebsiella pneumoniae*, resistance is most often driven by KPC-type carbapenemases, while *Escherichia coli* and *Enterobacter* species increasingly carry NDMs [6,7]. *Pseudomonas aeruginosa* typically demonstrates resistance via inactivation or mutation of the OprD porin and overexpression of the MexAB–OprM efflux system, rather than carbapenemase production alone [8]. This species-specific distribution of resistance underscores the importance of timely and precise differentiation between carbapenem-resistant (CR) and carbapenem-susceptible (CS) strains to guide therapy and infection control strategies.

In addition, alterations in outer membrane permeability, often due to the loss or modification of porin proteins (e.g., OmpK35, OmpK36), reduce the entry of antibiotics into the bacterial cell. Concurrently, the upregulation of efflux pump systems (such as AcrAB-TolC or MexAB-OprM) can actively expel carbapenems and other antibiotics, further contributing to resistance. These mechanisms often act synergistically, particularly in Gram-negative bacteria, leading to high levels of resistance and limited therapeutic options [9]. This rising resistance poses a serious obstacle to the management of severe infections [7,10], highlighting the importance of timely microbiological testing and comprehensive interpretation of the antibiogram to guide appropriate, targeted therapy and prevent the escalation of complicated infections.

Clinically, distinguishing between CR and CS MDR strains is essential for guiding empirical therapy and selecting appropriate antibiotics. Effective management of MDR UTIs relies heavily on the timely identification of resistance mechanisms, along with robust infection control measures within healthcare facilities [11]. Early recognition of whether a MDR strain is CR or CS is critical, as it directly informs therapeutic decisions and enables a more targeted treatment approach [12].

Although numerous studies have identified general risk factors for UTIs caused by CR microorganisms—such as residence in long-term care facilities, prolonged hospitalization, urinary catheter use, impaired functional status, and prior exposure to carbapenems [13,14]—there remains a lack of data differentiating risk profiles between CR and CS MDR strains in a comparative framework. Most existing research focuses on CR infections in isolation, without assessing how they differ from other MDR cases that remain carbapenem-susceptible. This study addresses that gap by directly comparing clinical and microbiological characteristics between CR and CS MDR UTI isolates. By doing so, it aims to identify distinguishing risk factors and outcomes associated specifically with carbapenem resistance, offering new insights that can inform empirical treatment strategies and targeted prevention measures in high-risk patient populations. Additional research has highlighted other risk factors, such as the presence of urinary catheters, recent surgical procedures, intensive care unit (ICU) stays, and immunosuppression [15,16]. Conditions like dementia, heart failure, and connective tissue diseases have also been implicated [17]. A high ASA score, immunosuppression, the presence of indwelling urinary catheters, and prolonged postoperative catheterization exceeding 48 hours were identified as specific risk factors for MDR UTIs—including CR strains—in urological settings [18]. Furthermore, risk factors associated with mortality in patients with CR UTIs include age over 60 years, septic shock, and dialysis [19].

Nevertheless, many of these factors are common to MDR UTIs in general and are not necessarily specific to carbapenem-resistant infections [16]. Previous studies investigating pathogen profiles have reported variability in the distribution of Enterobacterales including differing proportions of *Klebsiella pneumoniae*, *Escherichia coli*, and *Pseudomonas aeruginosa*, [2,4,5]. Similarly, the prevalence of specific carbapenemases among these organisms has shown considerable variation across reports [20,21,22].

The aim of this study is to perform a comparative analysis of MDR strains involved in UTIs, specifically differentiating between those resistant and those susceptible to carbapenems. The primary objective is to identify risk factors associated with UTIs caused by CR MDR pathogens, in comparison to those related to MDR infections caused by pathogens that retain susceptibility to carbapenems. By analyzing demographic, clinical, and microbiological data, the study seeks to evaluate and contrast the role of these risk factors in the development of both categories of MDR UTIs.

Additionally, the study aims to assess the impact of these risk factors on clinical outcomes, including disease severity, complication rates, length of hospital stay, and mortality. It also explores the bacterial species involved, their antimicrobial susceptibility profiles, and the presence of carbapenemase enzymes contributing to resistance. A better understanding of these risk factors could enhance early identification and prevention strategies by characterizing patients at elevated risk. Furthermore, detailed knowledge of the pathogens and their resistance patterns may support more effective management through optimized antibiotic selection and timely surgical intervention when necessary.

## 2. Results

Between 1 October 2023, and 31 March 2025, we conducted a retrospective comparative study involving two patient cohorts diagnosed with UTIs caused by MDR pathogens: one group infected with CR strains (N = 127) and another with CS isolates (N = 91).

Demographic analysis (Table 1) revealed no statistically significant differences between the CR and CS groups in terms of gender distribution (*p* = 0.14), mean age (*p* > 0.05), or place of residence (*p* = 0.96). Although the CR group had a slightly higher number of male patients and urban residents, these differences were not statistically meaningful.

The anatomical localization of the UTIs—upper versus lower urinary tract and right versus left side—also showed no significant intergroup variation. Upper tract involvement was noted in 70.86% of the CR group and 65.93% of the carbapenem-susceptible group (*p* = 0.53), while lower tract infections occurred in 29.14% and 34.07%, respectively. Laterality analysis revealed right-sided involvement in 53.54% of cases with resistant strains and 45.05%with susceptible strains (*p* = 0.27); left-sided infections were present in 46.45% and 54.95%, respectively.

In terms of comorbidities, the prevalence of neoplasia (*p* = 0.002), neurological disorders such as stroke sequelae (*p* < 0.001), and the presence of urosepsis at the time of admission (*p* < 0.001) were significantly higher among patients with carbapenem-resistant UTIs. This group also experienced significantly higher in-hospital mortality (*p* = 0.004) and a greater incidence of septic shock (*p* = 0.02). Other conditions, including type 2 diabetes mellitus, chronic kidney disease, heart failure, anemia, hypertension, obesity, and transfer from external healthcare facilities, did not differ significantly between the two groups. Detailed comparative data are presented in Table 1.

Analysis of urinary catheter presence at the time of diagnosis revealed a significantly higher prevalence in the carbapenem resistant group (88.98%) compared to the carbapenem-susceptible group (72.52%) (*p* = 0.003). The use of permanent nephrostomy catheters was also significantly more common among patients with resistant infections (24.40% vs. 10.31%, *p* = 0.02). Conversely, the proportion of patients without any urinary catheter in place at the time of diagnosis was notably higher in the carbapenem-susceptible group (27.47% vs. 11.02%, *p* = 0.003).

There were no statistically significant differences between the groups regarding the use of other types of catheters, including permanent urethral catheters, permanent double J ureteral catheters, double J ureterostomy catheters, or cystostomy tubes. Similarly, the duration of permanent urinary catheter use (<1 month vs. >1 month) did not differ significantly between groups. Further details are provided in Table 2.

Analysis of risk factors associated with MDR carbapenem-resistant urinary tract infections revealed significantly higher rates of prior hospitalization within the previous 180 days in the resistant group compared to the susceptible group (76.37% vs. 53.84%, *p* < 0.001). Similarly, recent antibiotic use during the same period was significantly more frequent among patients with CR infections (67.71% vs. 40.65%, *p* < 0.001). The insertion or replacement of percutaneous nephrostomy tubes was also notably higher in this group (24.40% vs. 8.79%, *p* = 0.005).

Furthermore, the overall proportion of patients who underwent any urological procedure prior to the onset of MDR infection was significantly greater in the CR group (88.97% vs. 71.42%, *p* = 0.001), and also the previous treatment with carbapenems (14.17% vs. 0%, *p* < 0.001).

No statistically significant differences were observed between the two groups regarding ICU admission, or specific urological procedures such as transurethral resection of the prostate (TURP), transurethral resection of bladder tumors (TURBT), urethral catheter insertion or replacement, percutaneous nephrolithotomy (PCNL), double J catheter insertion or replacement, open surgical procedures, ureterostomy stent replacement, cystostomy, or retrograde intrarenal surgery (RIRS). Detailed findings are presented in Table 3.

Multivariate logistic regression analysis identified several independent predictors of carbapenem resistance. These included the presence of neurological disorders (OR = 7.427; 95%CI: 2.804–19.674; *p* < 0.0001), neoplasia (OR = 2.152; 95%CI: 1.044–4.436; *p* = 0.038), prior antibiotic treatment within the previous 180 days (OR = 2.792; 95%CI:1.487–5.396; *p* = 0.001), and previous use of carbapenems (OR = 10.313;95% CI: 1.277–83.248; *p* = 0.029) (Table 4).

The model demonstrated good predictive performance, with a sensitivity of 82.5%, specificity of 54.6%, and an overall accuracy of 70%. These results indicate its potential clinical utility in identifying patients at increased risk for MDR urinary tract infections caused by carbapenem-resistant pathogens.

An additional analysis assessed the length of hospital stay between the two groups. Patients with MDR UTIs caused by CR pathogens had a significantly longer mean hospitalization duration (10.56 ± 10.95 days) compared to those with CS strains (4.28 +/− 5.5 days). This difference was statistically significant (Mann–Whitney test, *p* < 0.0001), highlighting the considerable clinical and economic burden associated with carbapenem-resistant infections. Further details are provided in Table 5.

The distribution of bacterial species isolated from urine cultures differed significantly between the two study groups. *Klebsiella* spp. was the most frequently identified pathogen in the CR group (71.65%), compared to 41.75% in the CS group. *Pseudomonas aeruginosa* was also notably more prevalent among resistant isolates (21.25% vs. 1.09%). In contrast, *Escherichia coli* was a frequently isolated in the CS group (39.56%) but was not detected in the CR group.

Other bacterial species, including *Proteus* spp. and *Staphylococcus* spp., were found exclusively in the CS group, whereas *Acinetobacter,* and *Enterobacter* species and *Morganella morgagnii* were isolated only in the CR group. These patterns suggest that specific pathogens—particularly non-fermenting Gram-negative bacilli and carbapenemase-producing organisms—are more commonly associated with MDR infections exhibiting carbapenem resistance. Further details are provided in Table 6.

Comparison of antibiotic resistance profiles between the two groups revealed statistically significant differences for the majority of tested agents. The CR group exhibited markedly higher resistance rates across most antibiotics, with the exception of ampicillin, trimethoprim/sulfamethoxazole, and amikacin, for which no significant intergroup differences were observed (*p* > 0.05).

The most pronounced disparities in resistance were noted for nitrofurantoin, fluoroquinolones (ciprofloxacin and levofloxacin), cephalosporins (cefuroxime, ceftriaxone, ceftazidime, and cefepime), and β-lactam/β-lactamase inhibitor combinations such as piperacillin/tazobactam. Resistance to colistin and fosfomycin was also significantly more frequent in the CR group.

Notably, all isolates in the resistant group demonstrated resistance to both imipenem and meropenem. Furthermore, pandrug resistance was observed exclusively within this group, affecting 14.84% of isolates.

Detailed resistance profiles are presented in Table 7.

Among the 60 patients tested for carbapenemase production, a total of 79 carbapenemase enzymes were identified, indicating that multiple enzyme types were present in several isolates. The most frequently detected enzyme was New Delhi metallo-β-lactamase (NDM), found in 48.15% of cases, followed by *Klebsiella pneumoniae* carbapenemase (KPC) in 29.63% and oxacillinase (OXA−48) in 13.58%. Verona integron-encoded metallo-β-lactamase (VIM) and imipenemase (IMP) were each detected in a single patient.

Notably, 33.80% of patients harbored two distinct carbapenemases, suggesting a high level of multidrug resistance and severely constrained therapeutic options. Only one patient tested negative for all five carbapenemases, indicating that nearly all cases of carbapenem resistance were enzyme-mediated. These findings underscore the predominance of NDM and KPC as the primary resistance mechanisms in this cohort. Full distribution data are presented in Table 8.

We identified only one *Morganella morganii* isolate in the CR group that tested negative for carbapenemase production; this isolate was classified as PDR, exhibiting non-susceptibility to all tested antimicrobial agents across all categories.

### Carbapenemase Expression by Bacterial Species 

**Figure 1:**
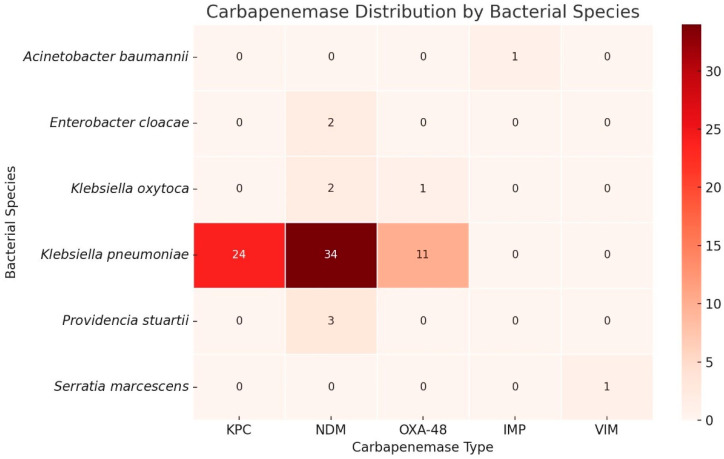
Distribution of Patients by Bacterial Species and Type of Carbapenemase.

Figure 1 details the distribution of carbapenemase enzymes across various bacterial species isolated from patients with carbapenem-resistant urinary tract infections. The most significant contributor to carbapenemase-mediated resistance was *K. pneumoniae*, which accounted for 51 of the 59 enzyme-positive cases. Among these, NDM (New Delhi metallo-β-lactamase) was the most prevalent enzyme (detected in 34 isolates), followed by KPC (*Klebsiella pneumoniae* carbapenemase) in 24 isolates and OXA-48 in 10. Notably, only *Klebsiella* spp. (particularly *K. pneumoniae* and *K. oxytoca*) demonstrated co-expression of two carbapenemase types—observed in 20 patients—which indicates a higher adaptive capacity and a more complex resistance profile in this genus.

## 3. Discussion

Understanding the epidemiology of UTIs caused by MDR organisms—especially carbapenem resistant strains—is essential for improving patient management and tailoring treatment strategies. In our study, patients in both groups were predominantly over the age of 65, had upper UTIs, and presented with multiple comorbidities, including diabetes, neoplasia, chronic kidney disease, anemia, stroke, hypertension, and obesity. However, patients in the carbapenem-resistant group had significantly higher rates of urosepsis and septic shock upon admission, as well as increased mortality. This group also exhibited a higher prevalence of urinary catheter use, particularly nephrostomy catheters.

Additionally, patients with carbapenem-resistant MDR UTIs had more frequent hospitalizations in the previous 180 days, recent antibiotic treatments, and a greater number of urological procedures, particularly for nephrostomy catheter insertion or replacement. Multivariate logistic regression identified neurological disorders, malignancies, antibiotic use in the past 180 days, and prior exposure to carbapenems as independent risk factors for the development of carbapenem-resistant MDR UTIs. While the predictive model demonstrated satisfactory sensitivity in identifying patients at risk for carbapenem-resistant UTIs, its specificity was relatively low (54.6%). This indicates a substantial rate of false positives, meaning that a notable proportion of patients without CR infections may be misclassified as high risk. In clinical practice, this could lead to overuse of broad-spectrum or last-line antibiotics such as carbapenems or colistin, potentially exacerbating resistance trends. However, in high-stakes infections such as CR UTIs—where delays in appropriate therapy can significantly worsen outcomes—favoring sensitivity over specificity may be justifiable.

*K. pneumoniae* and *P. aeruginosa* were the predominant pathogens in the CR group, accounting for over 90% of infections. Bacterial strains in this group exhibited significantly higher resistance to nearly all antibiotics tested, with the exception of trimethoprim/sulfamethoxazole. Notably, all but one isolate in the CR group tested positive for at least one carbapenemase enzyme, most frequently NDM and KPC.

Analysis of demographic data revealed similarities between the two groups, with relatively equal proportions of male and female patients—contrary to other studies where either males [23] or females [24] predominated. As expected, the majority of patients were older than 65 years in both groups, reflecting the increased incidence of urological and nephrological conditions with advancing age. However, age did not emerge as a significant risk factor for developing CR-UTIs, unlike findings from previous studies [25].

In contrast to a previous study conducted in our clinic, which reported a higher incidence of CR-UTIs among patients from rural areas [26], our current findings show an equal distribution between rural and urban residents in both groups. Upper urinary tract infections were more frequent in both groups, suggesting an elevated risk for complications such as sepsis, septic shock, and death. Notably, the incidence of urosepsis, septic shock, and mortality was significantly higher in the CR-UTI group, emphasizing the greater virulence of carbapenem-resistant pathogens. These findings differ from some earlier reports that observed similar mortality rates between resistant and susceptible strains [13], though our observed mortality rate remains lower than those reported in other studies [27,28].

Although a larger proportion of patients in the CR-UTI group were transferred from other hospitals, this difference was not statistically significant. This suggests that carbapenem resistance alone may not have been the primary reason for patient transfer.

The vast majority of patients in both groups had multiple comorbidities, with diabetes, chronic kidney disease, cardiovascular conditions, anemia, hypertension, and obesity occurring at similar rates across both groups. Although previous studies have identified diabetes, heart disease, and kidney failure as risk factors for carbapenem resistant UTIs [29], our findings did not support this association. Instead, we observed that certain conditions—specifically neoplasia and neurological disorders—were more frequently associated with CR UTIs. The strong association between neurological disorders and CR UTIs may be multifactorial. Patients with neurological impairments often experience decreased mobility, bladder dysfunction (e.g., neurogenic bladder), and are more likely to require chronic catheterization—all of which increase the risk of both colonization and infection with multidrug-resistant organisms. Furthermore, prolonged hospitalization, and reduced immune response may contribute to both acquisition and progression of infection. These factors may explain why neurological disorders emerged as an independent risk factor in our cohort.

The presence of urinary catheters such as urethral catheters, double J stents, and nephrostomy catheters is a well-established risk factor for MDR UTIs [4,17,18,19]. Therefore, we investigated whether these devices also contribute to the risk of carbapenem resistance. Our data showed that only permanent nephrostomy catheters were significantly more prevalent in the CR UTI group compared to the control group. However, the overall number of patients using various types of urinary catheters was comparable between the groups. This suggests that while catheterization plays a critical role in the development of MDR UTIs, it may not be a specific risk factor for carbapenem resistance.

In univariate analysis, several additional risk factors were more frequently observed in the CR UTI group: hospitalization within the past 180 days, antibiotic use in the past 180 days, previous carbapenem therapy, percutaneous nephrostomy insertion or replacement, and prior urological procedures. Except for prior carbapenem treatment, these are recognized risk factors for MDR UTIs [5,11,20], and were found in a majority of patients in both study and control groups. In particular, patients undergoing urological procedures who remain catheterized—especially those with nephrostomy tubes—are at high risk for MDR UTIs. Among these, those previously treated with carbapenems appear at elevated risk for developing CR UTIs. However, only 14.06% of patients in the CR group had a history of carbapenem treatment, suggesting that other mechanisms likely contribute to the emergence of carbapenem resistance.

Multivariate analysis of variables that significantly differed between the two groups identified four major independent risk factors associated with MDR CR UTIs compared to MDR CS UTIs: the presence of neurological disorders, neoplasia, recent antibiotic therapy within the past 180 days, and prior treatment with carbapenems. The odds ratio for prior carbapenem treatment was notably high (OR = 10.313), but the associated confidence interval was extremely wide (95% CI: 1.277–83.248), indicating substantial statistical uncertainty. This likely reflects the small number of patients with documented prior carbapenem exposure in the cohort, which reduces the stability of the estimate in logistic regression.

The association between neoplasia and CR UTIs, also observed in other studies [2,26,30], may be attributed to the immunosuppressed status of oncology patients, which increases their susceptibility to colonization or infection with more aggressive, hospital-acquired pathogens.

Neurological disorders—most often post-stroke sequelae—emerged as a somewhat unexpected risk factor and remain difficult to explain definitively. Reduced mobility and compromised personal hygiene in these patients may contribute to an increased risk of infection. Prospective studies focusing specifically on this factor are warranted to elucidate the underlying mechanisms. While many studies have highlighted comorbidities in general as risk factors for CR UTIs [4,12,16], few—if any—have singled out neoplasia and neurological impairment as the only comorbid predictors. The link between neurological disorders and CR UTIs may be due to associated risk factors such as immobility, incontinence, and catheter use. These conditions often require caregiver support and may compromise hygiene, increasing colonization risk. While speculative, this hypothesis is supported by patterns observed in older or functionally dependent populations. Further research is needed to explore the causal pathway between neurological dysfunction and UTI risk, particularly studies that integrate functional status, catheter management, and caregiver-dependency data. Prospective cohort studies or risk modeling incorporating these variables could help refine prevention strategies in neurologically impaired populations.

Recent antibiotic therapy (excluding carbapenems) in the past 180 days is a well-documented risk factor for MDR infections and, in our study, was also significantly associated with CR UTIs—an observation consistent with previous findings [11,12]. This may reflect the cumulative selective pressure imposed by repeated antibiotic exposure in patients with recurrent UTIs.

Prior treatment with carbapenems was, as expected, a significant predictor of carbapenem resistance, aligning with previously reported data [31], although some studies have not confirmed this relationship [32]. Recognizing and addressing these risk factors can aid in reducing unnecessary carbapenem use and inform the development of targeted treatment and prevention strategies. These findings carry important implications for managing nosocomial UTIs and implementing effective hospital antibiotic stewardship programs.

Finally, the successful control of MDR UTIs also depends on robust infection prevention protocols, particularly in high-risk areas such as intensive care units and among critically ill patients, as emphasized in recent research [23,24].

The longer duration of hospitalization observed in the study group is, to some extent, expected, given the presumed higher virulence of CR pathogens.

When analyzing the distribution of etiologic agents, both groups demonstrated a similar range of diversity, with eight different bacterial species identified in each. However, the composition differed slightly. In line with previous studies [5,25,26], the vast majority of isolates in both groups were *Klebsiella* spp. Notably, in our study, *Klebsiella* spp. emerged as the most frequent pathogen associated with carbapenem resistance, accounting for 71.65% of all CR isolates.

A particularly noteworthy finding was that all pathogens identified in the CR group belonged to the Enterobacterales order, which aligns with existing literature indicating that these organisms are the most frequent causative agents of UTIs [33]. Among them, *Klebsiella* spp. and *Pseudomonas aeruginosa* were dominant, jointly responsible for 92.9% of CR UTIs. This highlights the importance of continuous microbiological surveillance and rapid adaptation of empirical antibiotic regimens [28,29].

Previous studies have reported a wide range of CR pathogens. For example, alongside *Klebsiella* spp., some investigations identified *Enterobacter*, *Escherichia coli*, and *Providencia stuartii* [32]; others reported *Klebsiella pneumoniae*, *Citrobacter freundii*, *Enterobacter* spp., and *E. coli* [34]. In contrast, some studies found *Pseudomonas* spp. to be the most frequent isolate (23.5%), followed by *E. coli* and *K. pneumoniae*.

Compared to these findings, our study also identified less frequently reported species such as *Acinetobacter* spp., *Serratia marcescens*, and *Morganella morganii*. The prominence of carbapenem-resistant Enterobacterales (CRE), particularly carbapenem-resistant *Klebsiella pneumoniae*, as major contributors to morbidity and mortality underlines the urgent need for improved therapeutic strategies and infection control measures [31,32].

When analyzing the antibiotic resistance spectrum of microorganisms isolated from the two groups, we observed near-universal resistance to ampicillin in both, consistent with findings from previous local studies [35]. For the remaining tested antibiotics—excluding trimethoprim/sulfamethoxazole—the CR group demonstrated significantly higher resistance rates compared to the CS group, particularly to fluoroquinolones and cephalosporins, as also reported in other studies [33].

Notably, resistance to colistin—an antibiotic often used as a last resort for CR UTIs—was observed in 25.98% of isolates in the CR group. This relatively high resistance rate may be attributed to the increasingly frequent use of colistin in our hospital over recent years, in response to a growing number of CR UTI cases. Resistance levels, however, vary significantly across regions. Some European surveillance studies have reported colistin resistance rates as low as 1.1% in Spain [36], likely due to stricter stewardship and limited use of colistin. In contrast, studies from Italy have documented much higher rates, reaching up to 38% [37], often in settings where colistin is used empirically and access to susceptibility testing is limited. Our findings appear to fall between these extremes and highlight the urgent need for local stewardship protocols and routine susceptibility testing to guide appropriate colistin use.

A positive finding from our analysis is the very low resistance rate to amikacin in the CR group, only 3.91%, which closely aligns with another study reporting a resistance rate of 4.3% [38]. However, this contrasts sharply with other research showing markedly higher resistance to amikacin, as high as 64.1% [23]. One plausible explanation is the relatively infrequent use of amikacin in our hospital’s empirical treatment protocols, which may have reduced selective pressure for resistance. Unlike other aminoglycosides, amikacin is less susceptible to common aminoglycoside-modifying enzymes, which may also contribute to its retained activity. Furthermore, clinicians may reserve amikacin for severe infections due to concerns about nephrotoxicity, which limits its overuse. These factors combined may explain the comparatively lower resistance rates observed in our setting.

Recent Romanian studies confirm that *E. coli* and *Klebsiella pneumoniae* are the main MDR uropathogens. In southeastern Romania, *E. coli* showed high resistance to fluoroquinolones and cephalosporins [2], while pediatric data also revealed frequent MDR among *E. coli* and *Klebsiella* spp. [39]. Female cohorts demonstrated significant resistance to aminopenicillins, fluoroquinolones, and cephalosporins, with carbapenems and aminoglycosides retaining activity [40,41] Surveillance from Bucharest confirmed a rising trend of resistance over five years [42], and hospital-based studies highlighted the emergence of carbapenem-resistant *K. pneumoniae*, often linked with poor outcomes [43,44,45].

Beyond pathogen profiles, MDR UTIs in Romania and the Balkans are associated with longer hospital stays, increased costs, and limited therapeutic options [46]. Although not the main focus here, it is worth mentioning that pandemic-related pressures may have contributed to worsening resistance, including the first Romanian report of mcr-mediated colistin resistance. Collectively, these data highlight a consistent rise in MDR UTIs, dominated by *E. coli* and *Klebsiella*, with carbapenem resistance representing the most serious threat. As a tertiary referral center, our clinics manage the most severe and complex cases, including a high number of patients with sepsis who require last-resort antibiotics. This clinical context inherently increases the risk of selecting for Enterobacterales strains resistant to advanced-generation antimicrobials. In recent years, we have encountered a growing number of MDR UTIs that are susceptible only to carbapenems, which has led to a sharp increase in the use of meropenem and imipenem.

While this intensified use of carbapenems initially contributed to improved survival in patients with sepsis—particularly urosepsis—it has also resulted in the unintended consequence of rising ESBL production and, subsequently, the emergence of carbapenem-resistant strains. This shift poses significant challenges in updating and implementing treatment guidelines, especially in selecting effective empirical antibiotics.

Moreover, our findings indicate a notable prevalence of pan-drug-resistant bacteria in the carbapenem-resistant group, which likely contributes to the significantly higher mortality observed in these patients. PDR infections represent the extreme end of antimicrobial resistance, where no effective antibiotic options remain, often leaving clinicians with limited or purely supportive care strategies. This therapeutic dead-end drastically increases the risk of treatment failure, sepsis progression, and death. The presence of such pathogens underscores a critical and immediate need for robust antibiotic stewardship, improved infection control measures, and the accelerated development and implementation of novel antimicrobial agents or adjunctive therapies.

Our study suggests that reducing overall antibiotic use—potentially by minimizing the duration of urinary catheterization—may help limit the development of antimicrobial resistance. Some authors have proposed withholding carbapenems in cases of carbapenem-susceptible UTIs, reserving their use for critically ill patients [45,47]. Instead, they suggest administering alternative antibiotics to which the pathogen may show some resistance. However, we believe this approach carries a substantial risk of progression to sepsis, especially considering that most UTIs in our cohort were complicated infections occurring in postoperative patients with urinary catheters.

Gardner et al. conducted a thorough narrative review of antimicrobial stewardship, focusing specifically on carbapenem de-escalation among hospitalized patients, including those with UTIs. They highlighted that for non–critically ill, carbapenem-susceptible infections, transitioning to narrower-spectrum agents is both safe and effective—and that withholding carbapenems until absolutely necessary can reduce unnecessary broad-spectrum exposure without compromising outcomes [48]. Moreover, recent stewardship analyses suggest that withholding carbapenems in CS UTIs is safe in non-critically ill patients, with successful de-escalation achievable in over 40% of cases without adverse outcomes [49].

Non-carbapenem antibiotics such as piperacillin/tazobactam have been proposed as effective alternatives in moderate infections caused by ESBL-producing bacteria, offering the potential to delay or reduce carbapenem resistance and preserve the efficacy of these last-line agents [50]. These considerations underscore the critical need for therapeutic protocols guided by regularly updated local susceptibility patterns, ensuring both safety and effectiveness in clinical decision-making.

In the subset of 60 patients in whom carbapenemase testing was performed, 59 showed expression of at least one carbapenemase enzyme. This finding confirms that carbapenem resistance in our region is primarily enzyme-mediated, with *New Delhi metallo-beta-lactamase* (NDM), *Klebsiella pneumoniae carbapenemase* (KPC), and OXA-48 being the most frequently detected, consistent with previous reports [3,6,8,50].

Additionally, we observed a relatively high percentage (over 33%) of patients whose bacterial isolates expressed two different carbapenemase enzymes—comparable to findings from some studies [20], but substantially higher than the 5% reported in others [22]. This high rate of co-expression may indicate increasing genetic complexity and further complicate treatment, reinforcing the need for ongoing surveillance and tailored antibiotic policies.

Our study has several limitations. The retrospective design is a primary constraint, as we could not anticipate the surge in carbapenem-resistant UTIs in our clinics to enable a prospective approach. Another limitation is that carbapenemase testing was performed on only 60 of the 127 CR isolates, due to logistical and resource constraints. Although the tested subset was selected to reflect the broader isolate population, it is possible that certain resistance mechanisms or carbapenemase variants were underrepresented. This limits our ability to draw definitive conclusions about the overall distribution of carbapenemase genes in the study population. Additionally, the single-center nature of the study may restrict the generalizability of our findings to broader populations. However, it is important to note that many patients were referred from other hospitals across the region. As a result, our data likely reflect the regional burden of severe and complex cases, particularly those managed in specialized urology and nephrology departments.

Future prospective, multicenter studies are needed to strengthen the level of evidence supporting the risk factors identified in our study. Expanding the research across multiple institutions and patient populations will allow for broader validation and generalizability of these findings.

In the long term, research efforts should prioritize a deeper understanding of bacterial resistance mechanisms and the development of novel therapeutic strategies aimed at preventing and effectively treating severe MDR infections. Furthermore, assessing the economic burden associated with managing MDR infections is essential to inform the design of sustainable public health policies and antimicrobial stewardship programs.

## 4. Materials and Methods

This retrospective, observational study included all consecutive patients with culture-confirmed MDR UTIs hospitalized between 1 October 2023, and 31 March 2025, at the “Dr. C.I. Parhon” Tertiary Hospital in Iași, the largest specialized center for kidney diseases in northeastern Romania. Eligible patients were those admitted to the departments of urology, nephrology, internal medicine, geriatrics, and the intensive care unit (ICU). Only the first UTI episode per patient during the study period was included to avoid duplication. The study was approved by the hospital’s ethics committee (approval number: 1506/14.02.2025) and aimed to compare patients with culture-confirmed UTIs caused by MDR pathogens, which were grouped according to their susceptibility to carbapenems into carbapenem-resistant (CR) and carbapenem-susceptible (CS) categories.

In accordance with the European Association of Urology (EAU) Guidelines on Urological Infections [51], we defined UTIs as infections involving any part of the urinary tract, confirmed by clinical symptoms such as dysuria, frequency, urgency, flank pain, or fever, together with microbiological evidence of significant bacteriuria (≥10^5^ CFU/mL). We classified UTIs into localized and systemic forms. Localized UTIs referred to infections confined to the lower urinary tract, without systemic involvement (e.g., uncomplicated cystitis), while systemic UTIs included those associated with fever, chills, or sepsis (e.g., pyelonephritis, urosepsis), regardless of anatomical location. Catheter-associated UTIs were defined as symptomatic UTIs occurring in patients with an indwelling urinary catheter in place for ≥48 hours prior to symptom onset. Complicated UTIs were characterized by factors such as urinary tract obstruction, presence of foreign bodies (e.g., stents, catheters), immunosuppression, or anatomical abnormalities, which increased the risk of treatment failure or recurrence.

The study population was divided into two groups: one group consisting of patients with MDR UTIs caused by carbapenem-susceptible strains, and the other group including patients infected with carbapenem-resistant organisms. Strains were defined as resistant if resistance to at least one carbapenem antibiotic was documented. Patient data were extracted from the hospital’s electronic medical records using pathogen-specific ICD-10 codes: B96.1 (*Klebsiella pneumoniae*), B96.2 (*Escherichia coli*), B96.4 (*Proteus* spp.), B96.5 (*Pseudomonas* spp.), and B96.88 (other specified bacteria).

**Figure 2:**
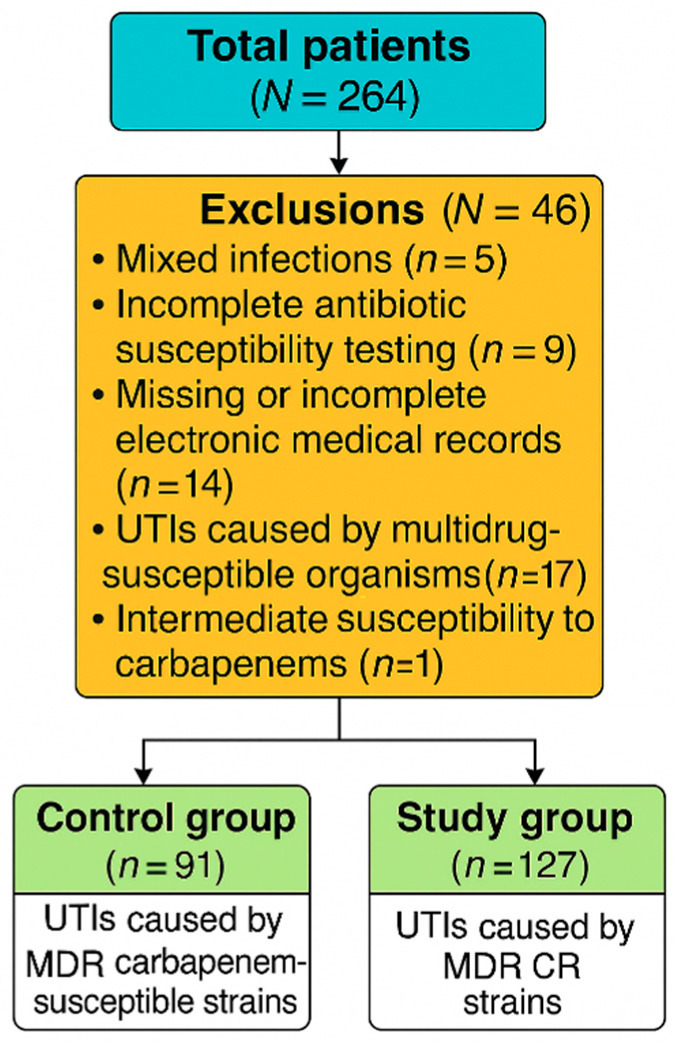
Flow diagram of patient selection.

Out of the eligible patients, 91 were assigned to the control group (MDR UTIs caused by carbapenem-susceptible strains), while 127 patients were included in the study group (MDR UTIs caused by CR strains). A total of 46 patients were excluded from the final analysis: 5 due to mixed infections, 9 due to incomplete antibiotic susceptibility testing, 14 due to missing or incomplete electronic medical records, and 17 with UTIs caused by multidrug-susceptible organisms. Additionally, one patient with a UTI caused by an isolate showing intermediate susceptibility to carbapenems was excluded (Figure 2).

Cases of urosepsis were identified according to the Sepsis-3 International Consensus criteria [20].

### 4.1. Microbiological Methods

UTI diagnosis was based on both clinical signs and a positive urine culture, defined as ≥10^5^ colony-forming units (CFU)/mL. Clinical symptoms included pollakiuria, urgency, pyuria, dysuria, fever, lumbar pain, and leukocytosis. Antimicrobial susceptibility testing was primarily performed using the Kirby–Bauer disk diffusion method. For antibiotics where EUCAST does not provide zone diameter breakpoints, minimum inhibitory concentrations (MICs) were determined using broth microdilution, in accordance with EUCAST recommendations. Susceptibility results were interpreted according to EUCAST breakpoint tables version 13.0 (valid from 1 January 2023) and version 14.0 (valid from 1 January 2024), depending on the isolate processing date.

The antibiotic panel used for susceptibility testing included: ampicillin, amoxicillin-clavulanate, cefuroxime, ceftriaxone, ceftazidime, cefepime, piperacillin-tazobactam, ciprofloxacin, levofloxacin, imipenem, meropenem, gentamicin, amikacin, fosfomycin, nitrofurantoin, and trimethoprim-sulfamethoxazole.

### 4.2. Carbapenemase Testing and Antimicrobial Classification

For the purposes of this study, bacterial strains classified as ‘susceptible, increased exposure’ (I)—previously labeled as ’intermediate’—were excluded from the analysis. In our dataset, only one *E. coli* isolate was classified as ‘susceptible at increased exposure’ to meropenem; all other isolates were either fully susceptible or resistant.

Additionally, 60 CR isolates underwent testing for carbapenemase production using the NG-Test CARBA5 (NG Biotech, France), which identifies five major carbapenemases: KPC (*Klebsiella pneumoniae* carbapenemase), NDM (New Delhi metallo-beta-lactamase), OXA-48 (oxacillinase-48), VIM (Verona integron-encoded metallo-beta-lactamase), and IMP (imipenemase). Due to limited availability, testing was performed on 60 consecutive patients between 1 October 2023, and 31 January 2024.

### 4.3. Outcome Measures

Demographic data (age, gender, place of residence, infection laterality, and UTI classification) and relevant clinical variables were recorded. Comorbid conditions such as diabetes mellitus, chronic kidney disease, obesity, anemia, neurological disorders, and malignancies were documented. We also evaluated the presence of urosepsis or septic shock at admission, as well as whether patients had been transferred from other healthcare institutions.

Information was collected regarding the presence and type of urinary catheters at the time of diagnosis, as well as any prior urological interventions or procedures. Key outcome parameters included in-hospital mortality and total length of hospitalization.

Infection-specific data were also analyzed, including the identification of the causative pathogen (e.g., *Escherichia coli*, *Klebsiella pneumoniae*, *Pseudomonas aeruginosa*) and the corresponding antibiotic susceptibility profiles.

### 4.4. Statistical Analysis

All collected data were entered and analyzed using IBM SPSS Statistics for Windows, Version 24.0 (IBM Corp., Armonk, NY, USA). Quantitative variables were expressed as either mean ± standard. Student’s *t*-test was used for normally distributed variables, while the Mann–Whitney U test was applied for non-normally distributed variables. Categorical variables were presented as percentages and compared using either the Chi-square test or Fisher’s exact test, as appropriate. Multivariate logistic regression analysis was performed to identify independent predictors of carbapenem resistance.

## 5. Conclusions

The findings of this study emphasize the urgent clinical need to refine the management of MDR UTIs, particularly those caused by carbapenem-resistant organisms. The strong association between resistance and poor outcomes such as septic shock and mortality highlights the importance of early risk stratification and rapid diagnostic tools to guide empirical therapy. In clinical practice, patients with neurological comorbidities, recent antibiotic exposure, or urinary devices should be considered high-risk and managed with heightened vigilance.

From a research standpoint, future studies should explore the role of functional status, catheter management practices, and caregiver dependency in the emergence of resistance, particularly in neurologically impaired populations. Additionally, large-scale molecular surveillance is needed to map the evolving distribution of carbapenemase genes. These insights are critical to inform stewardship strategies, diagnostic prioritization, and the development of effective treatment algorithms. To translate these findings into clinical practice, targeted antimicrobial stewardship and early risk identification must be prioritized. Practical strategies include integrating electronic health record (EHR) alerts to flag patients with recent carbapenem use, neurological comorbidities, or indwelling urinary devices. Screening protocols at admission—particularly in high-risk wards like neurology and urology—can help identify carriers of resistant organisms. In parallel, regular audits of antibiotic prescribing, especially involving carbapenems and colistin, should be combined with clinician feedback and local antibiogram updates to guide empiric therapy. These interventions can improve treatment precision, minimize unnecessary broad-spectrum antibiotic use, and ultimately reduce resistance pressure within hospital settings.

To support timely decision-making, rapid diagnostic tools like multiplex PCR assays (e.g., for detecting NDM, KPC, OXA), lateral flow tests for carbapenemase enzymes, and MALDI-TOF MS with resistance marker panels offer promising solutions. Implementing such technologies can reduce diagnostic turnaround time, optimize empirical therapy, and help contain the spread of resistant strains within healthcare settings.

## Figures and Tables

**Table 1:** Characteristics of the two groups.

	MDR CR group (N = 127)	MDR CS group (N = 91)	*p*-value
Male N. (%)	78(61.41%)	46 (50.54%)	0.14 ^C^
Female N. (%)	49(38.58%)	45 (49.46%)	
Age (mean ± SD) Male	67.50 ± 15.18	70.83 ± 13.32	0.38 ^MW^
Age (mean ± SD) Female	66.82 ± 15.59	67.11 ± 12.68	0.92 ^MW^
Urban Residence (U) N. (%)	64 (50.39%)	47 (51.64%)	0.96 ^C^
Rural Residence (R) N. (%)	63 (49.61%)	44 (51.64%)	
Localization of UTI			
Upper UTI	90 (70.86%)	60 (65.93%)	0.53 ^C^
Lower UTI	37 (29.14%)	31 (34.07%)	
Right side	68 (53.54%)	41 (45.05%)	0.27 ^C^
Left side	59 (46.45%)	50 (54.95%)	
Comorbidities Nr.(%)			
Type 2 diabetes (DM)	37 (28.13%)	34 (37.36%)	0.25 ^C^
Neoplasia	47 (36.00%)	16 (17.58%)	0.002 ^C^
Kidney failure	99 (77.95%)	66 (72.52%)	0.446 ^C^
Heart failure	60(47.24%)	45 (49.45%)	0.85 ^C^
Anemia	97 (76.37%)	59 (64.83%)	0.87 ^C^
Stroke sequelae (neurological disorders)	40 (31.49%)	5 (5.15%)	<0.001 ^C^
Hypertension	96 (75.59%)	71 (78.02%)	0.79 ^C^
Obesity	26 (20.47%)	25 (25.77%)	0.29 ^C^
Urosepsis at the moment of admission	61 (48.03%)	23 (25.27%)	0.001 ^C^
Mortality	21(16.53%)	3 (3.09%)	0.004 ^C^
Septic shock	31 24.40%)	10 (10.98%)	0.02 ^C^
Transfer from other hospitals	37 (28.13%)	18 (18.55%)	0.15 ^C^

^MW^—Mann–Whitney test, ^C^—Chi-square test.

**Table 2:** Presence of urinary catheters at the time of diagnosis.

Catheter Type	MDR CR Group (N = 127)	MDR CS Group (N = 91)	*p*-Value
Urinary catheters at the time of diagnosis	113 (88.98%)	66 (72.52%)	**0.003 ^C^**
Permanent urethral catheter	41 (32.28%)	29 (31.86%)	0.93 ^C^
Permanent double J ureteral catheter	39 (30.70%)	25 (25.77%)	0.71 ^C^
Permanent nephrostomy catheter	31 (24.40%)	10 (10.31%)	**0.02 ^C^**
Permanent ureterostomy double J catheter	1 (0.78%)	1 (1.03%)	1.00 ^F^
Permanent cystostomy catheter	1 (0.78%)	1 (1.03%)	1.00 ^F^
Total permanent urinary catheters < 1 month	62 (48.81%)	40 (43.95%)	0.56 ^C^
Total permanent urinary catheters > 1 month	51 (40.15%)	26 (28.57%)	0.1 ^C^
Total patients without catheter at hospitalization	14 (11.02%)	25 (27.47%)	**0.003 ^C^**

^F^—Fisher test, ^C^—Chi-square test.

**Table 3:** Risk factors for the occurrence of MDR infections.

Risk Factor	MDR CR Group (N = 127)	MDR CS Group (N = 91)	*p*-Value
Hospitalization in the past 180 days	97 (76.37%)	49 (53.84%)	**<0.001 ^C^**
Antibiotherapy in the past 180 days (without carbapenems treatment)	86 (67.71%)	37 (40.65%)	**<0.001 ^C^**
ICU stay	32 (25.19%)	14 (15.38%)	0.11 ^C^
Previous history of carbapenems treatment	18 (14.17%)	0 (0%)	<0.001 ^C^
TURP (+/−lithotripsy)	7 (5.51%)	3 (3.29%)	0.52 ^F^
TURBT	6 (4.72%)	5 (5.49%)	0.95 ^C^
Percutaneous nephrostomy tube insertion/replacement	31 (24.40%)	8 (8.79%)	**0.005 ^C^**
Urethral catheter insertion/replacement	42 (33.07%)	31 (34.06%)	0.99 ^C^
PCNL	2 (1.57%)	1 (1.03%)	1 ^F^
Double J catheter insertion/replacement	40 (31.49%)	25 (25.77%)	0.62 ^C^
Open surgery	5 (3.93%)	2 (2.06%)	0.7 ^F^
Ureterostomy double J catheter replacement	1 (0.78%)	1 (1.03%)	1 ^F^
Cystostomy	1 (0.78%)	1 (1.03%)	1 ^F^
Total patients with urological maneuvers before MDR	113 (88.97%)	65 (71.42%)	**0.001 ^C^**
RIRS	4 (3.14%)	2 (2.06%)	1 ^F^

^F^—Fisher test, ^C^—Chi-square test; ICU—intensive care unit; TURP—transurethral resection of the prostate; TURBT—transurethral resection of bladder tumors; PCNL—Percutaneous nephrolithotomy; RIRS—retrograde intrarenal surgery.

**Table 4:** Multivariate logistic regression results–predictors of carbapenem resistance.

Predictor Variable	Odds Ratio (OR)	95% CI (Lower)	95% CI (Upper)	*p*-Value
Neurologic deficits	7.427	2.804	19.674	<0.0001
Neoplasia	2.152	1.044	4.436	0.038
Antibiotics in the past 180 days	2.792	1.487	5.396	0.001
Previous Carbapenem Treatment	10.313	1.277	83.248	0.029

CI—confidence interval.

**Table 5:** Comparison of hospitalization length by carbapenem resistance status.

Group	Mean (Days)	Median (Days)	Standard Deviation	N
Carbapenem-resistant	10.56	9	10.95	127
Carbapenem-susceptible	4.28	1	5.5	91

**Table 6:** Types of bacteria isolated in UTI MDR carbapenem-resistant vs. susceptible groups.

Type of Bacteria	UTI MDR CR (N = 127)	UTI MDR CS (N = 91)	Fisher Test (Value)
*Klebsiella* spp.	91 (71.65%)	38 (41.75%)	<0.001 *
*Escherichia coli*	0 (0%)	36 (39.56%)	<0.001
*Pseudomonas* spp.	27 (21.25%)	1 (1.09%)	<0.001
*Proteus* spp.	0 (0.00%)	12 (13.18%)	<0.001
Others	9 (7.08%)	4 (4.39%)	0.56 (not significant)

* Chi square test, *p*-value. Others" category includes the following species: UTI MDR CR group: *Acinetobacter* spp. (2), *Enterobacter* spp. (2), *Providencia* spp. (3), *Serratia marcescens* (1), *Morganella morganii* (1); UTI MDR CS group: *Providencia* spp. (1), *Serratia marcescens* (1), *Staphylococcus* spp. (2).

**Table 7:** Antibiotic resistance in the two groups (for gram-negative bacteria).

Antibiotic	CR (N = 127)	CS (N = 89)	*p* Value (Test)
Ampicillin	127 (100%)	88 (98.87%)	0.41 (Fisher’s exact) (not significant)
Amoxicillin/Clavulanate	127 (100%)	84 (95.45%)	0.011 (Fisher’s exact)
Trimethoprim/Sulfamethoxazole	60 (47.24%)	31 (34.83%)	0.09 (Chi-square) (not significant)
Nitrofurantoin	96 (75.59%)	33 (37.07%)	<0.001 (Chi-square)
Ciprofloxacin	120 (94.48%)	66 (74.15%)	<0.001 (Chi-square)
Levofloxacin	114 (89.76%)	64 (71.91%)	<0.001 (Chi-square)
Cefuroxime	96 (75.59%)	51 (57.30%)	<0.001 (Chi-square)
Ceftriaxone	96 (75.59%)	47 (52.80%)	<0.001 (Chi-square)
Ceftazidime	116 (91.33%)	45 (50.56%)	<0.001 (Chi-square)
Cefepime	117 (92.12%)	43 (48.31%)	<0.001 (Chi-square)
Piperacillin/Tazobactam	114 (89.76%)	7 (7.86%)	<0.001 (Chi-square)
Imipenem	127 (100%)	0 (0%)	<0.001 (Fisher’s exact)
Meropenem	127 (100%)	0 (0%)	<0.001 (Fisher’s exact)
Gentamicin	106 (83.46%)	38 (39.18%)	<0.001 (Chi-square)
Colistin	33 (25.98%)	2 (2.06%)	<0.001 (Fisher’s exact)
Fosfomycin	55 (43.30%)	1 (1.12%)	<0.001 (Fisher’s exact)
Amikacin	5 (3.93%)	1 (1.12%)	0.4 (Fisher’s exact) (not significant)
Pandrug-resistant isolates	19 (14.96%)	0 (0%)	<0.001 (Fisher’s exact)

Carbapenemase Distribution in Carbapenem -Resistant Isolates.

**Table 8:** Types of carbapenemases observed in the isolated specimens (59 patients).

Carbapenemase Type	Number of Enzymes	Percentage
KPC	24	30.38%
OXA	12	15.20%
NDM	41	51.90%
VIM	1	1.26%
IMP	1	1.26%
Patients with two enzymes detected	20	33.80%
Total number of enzymes in 59 patients	79	100%

## Data Availability

The original contributions presented in this study are included in the article. Further inquiries can be directed to the corresponding author.
